# Sustainability of the integrated chronic disease management model at primary care clinics in South Africa

**DOI:** 10.4102/phcfm.v8i1.1248

**Published:** 2016-11-17

**Authors:** Ozayr H. Mahomed, Shaidah Asmall, Anna Voce

**Affiliations:** 1 Public Health Medicine, University of KwaZulu-Natal, South Africa; 2 National Department of Health, South Africa

## Abstract

**Background:**

An integrated chronic disease management (ICDM) model consisting of four components (facility reorganisation, clinical supportive management, assisted self-supportive management and strengthening of support systems and structures outside the facility) has been implemented across 42 primary health care clinics in South Africa with a view to improve the operational efficiency and patient clinical outcomes.

**Aim:**

The aim of this study was to assess the sustainability of the facility reorganisation and clinical support components 18 months after the initiation.

**Setting:**

The study was conducted at 37 of the initiating clinics across three districts in three provinces of South Africa.

**Methods:**

The National Health Service (NHS) Institute for Innovation and Improvement Sustainability Model (SM) self-assessment tool was used to assess sustainability.

**Results:**

Bushbuckridge had the highest mean sustainability score of 71.79 (95% CI: 63.70–79.89) followed by West Rand Health District (70.25 (95% CI: 63.96–76.53)) and Dr Kenneth Kaunda District (66.50 (95% CI: 55.17–77.83)). Four facilities (11%) had an overall sustainability score of less than 55.

**Conclusion:**

The less than optimal involvement of clinical leadership (doctors), negative staff behaviour towards the ICDM, adaptability or flexibility of the model to adapt to external factors and infrastructure limitation have the potential to negatively affect the sustainability and scale-up of the model.

## Introduction

In 1994, South Africa adopted a primary health care (PHC) approach to health care delivery.^1^ Central to the implementation of PHC approach is the provision of a comprehensive community-based health service that includes preventive, promotive, curative and rehabilitative care.^1^ The clinic is the point of first contact for the patient or client and provides a one-stop service for at least 8 hours a day, 5 days a week.^2^ Clinics, as primary level services, are supported and strengthened by other levels of care including acute and specialised referral hospitals. The introduction of ward-based outreach teams and integrated school health teams as part of the PHC re-engineering initiative in 2010^3^ served to strengthen the community and health promotive aspects of PHC.

Although a concerted effort was made between 1994 and 2010 to promote the use of clinics as the primary contact point of health services, many patients bypassed the PHC clinics and attended hospitals for the initial contact visit, thereby increasing the cost of the service.^4^ Patients cited long queues of people, long waiting times, medication stock-outs, inadequate number of and inappropriately trained human resources and poorly structured and inaccessible PHC clinics^5^ as the main reasons for accessing higher levels of care.

The main contributing factors to the long queues and long waiting times include the vertical, disease-specific and curative nature of the service delivery.^6^ Patients with comorbidities are required to attend the facilities on multiple days depending on the number of conditions and the availability of service providers.^6^ Patients are provided with return dates for follow-up appointments based on their disease conditions and without prior consultation. Except for patients receiving antiretroviral treatment (ART) and anti-tuberculosis medication, there is no mechanism to track patients with non-communicable diseases (NCDs) who missed their appointments.^6^ Furthermore, the data collected at facility level are not being utilised for planning of services. Therefore, facility managers are unaware of the acute to chronic patient ratio and are unable to adjust medication stock levels, resulting in medication stock-outs.^6^

Human immunodeficiency virus (HIV) has been transformed into a chronic disease because of the widespread availability of ART. People with HIV are living longer and ageing, and are developing non-HIV-related chronic conditions similar to the rest of the population. Some NCDs are related to the HIV infection itself and to the side effects of some of the medicines used to treat HIV infection.^7^ The combination of increased life expectancy and availability and access to ART is likely to further increase the need for long-term care for the patients.

The current evidence suggests that chronic diseases especially NCDs are poorly detected, managed and monitored in the health system, and primary, secondary and tertiary prevention appear to be failing.^8^ As the burden of chronic diseases (both communicable and NCDs) increases, providing affordable and effective care to the often large and increasing numbers of people will be an immense challenge. Therefore, it is imperative that there is a reorientation of the services provided at PHC clinics from a curative focus to a more patient-centric approach.

From 2010, a renewed focus has been placed on strengthening the management of chronic conditions to increase life expectancy, as mandated by the National Service Delivery Agreement.^9^ The proposed strategies to targeting the management of chronic diseases include the reorganising and improving the functioning of clinical services and extending care for all chronic diseases (both communicable and NCDs) into communities, through an integrated approach, using the PHC re-engineering initiative.

**FIGURE 1:**
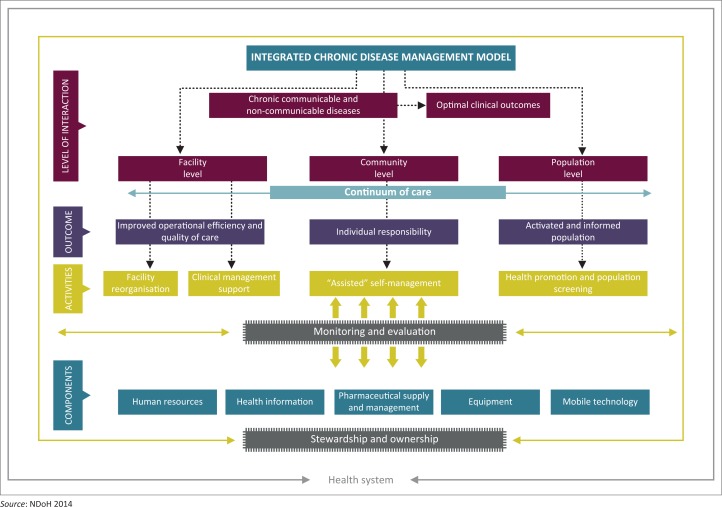
Integrated chronic disease management model for South Africa.

The National Department of Health (NDoH) initiated and implemented an integrated approach to the management of chronic diseases. The integrated chronic disease management (ICDM) model consists of four interrelated intervention phases – facility reorganisation, clinical supportive management, assisted self-supportive management and strengthening of support systems and structures outside the facility^10^ to ensure seamless transition of the patient from the facility to the community (Figure 1).

This study focuses on two components: facility reorganisation and clinical support management.

The facility reorganisation component is premised on the application of lean thinking principles of waste reduction and the 5-S system (sort, set in order, shine, standardise and sustain).^11^ Improved patient flow will be achieved through designation of service areas within the facility, integration of care for patients with chronic communicable and NCDs, the integration of clinical records, the standardisation of patient load through appointment scheduling for patients and scheduling of human resources to cater for the patient load and medication delivery through pharmacy courier or alternate delivery mechanisms such as adherence and support groups.^10^

Clinical management support focuses on improving the quality of clinical care provided to all chronic patients to achieve optimal clinical control of the disease and to decrease morbidity and mortality. Central to this is standardised clinical care based on national treatment protocols (clinical algorithms) that is supported by the introduction of a standardised chronic patient record to ensure that all steps in the holistic management of the chronic patients are followed.^10^

The initial implementation of the ICDM was facilitated by external technical assistance. A participatory approach to the implementation was adopted with both the senior technical advisor (SA) and project manager (OHM) visiting each facility to assist with the implementation and providing guidance on overcoming facility-specific obstacles.^12^ However, minimal or no additional resources other than the direct ICDM model requirements were provided. After a period of 12 months, the external facilitators withdrew their supportive supervisory visits.

Evidence suggests that even when initial implementation efforts are successful, interventions or programmes do not necessarily continue as originally implemented,^13^ with a failure rate of up to 70% reported.^14^ An unsustainable programme can waste money and resources, have a negative impact on the staff and community and may be detrimental to overall public health goals.^15^

In an attempt to increase the sustainability of health care improvement programmes for patients and health care services, the National Health Service (NHS) in the UK launched the NHS Sustainability Model (SM).^16^ The NHS SM consists of 10 factors relating to process, staff and organisational issues. The NHS SM has been designed for individual-planned improvement changes at a project level and not to determine sustainability at a general community or organisation level.

The aim of this study was to assess the implementation of the ICDM model 18 months post initiation and to determine its sustainability as a quality improvement process.

## Methods

### Study design

An observational cross-sectional study with an analytical component was conducted.

### Study setting

The ICDM was initiated between April 2011 and January 2013 at 42 PHC facilities across three districts: Dr Kenneth Kaunda District (DKK), North West Province; West Rand Health District (WRH), Gauteng Province; and Bushbuckridge (BBR) subdistrict within the Ehlanzeni District, Mpumalanga Province (Table 1).

**TABLE 1:** Profile of participating districts.

Province	Name of district	Population of district	Number of health care facilities	ICDM initiating sites
North West Province	Dr Kenneth Kaunda District	807 000 people	1 regional hospital, 3 district hospitals, 9 community health centres (CHCs), 27 fixed clinics, 6 satellite clinics, 2 mobile health service units^2^	10 facilities in DKK – 3 CHCs and 7 PHC clinics
Gauteng	West Rand Health District	900 000	60 health facilities: 1 regional hospital, 2 district hospitals, 4 CHCs, 39 PHC clinics^3^	15 facilities – 3 CHCs and 12 PHC clinics
Mpumalanga	Bushbuckridge subdistrict of Ehlanzeni District	BBR estimated population of 541 249	3 district hospitals, 2 CHCs, 36 fixed PHC clinics, 5 mobile clinics^4^	17 facilities – 2 CHCs and 15 PHC clinics

*Source*: District Profiles- Health Systems Trust

BBR, Bushbuckridge; DKK, Dr Kenneth Kaunda District; ICDM, integrated chronic disease management model; PHC, primary health care.

### Study population

The study population comprised all ICDM initiating facilities – 10 in DKK, 15 in WRH and 17 in BBR. Five facilities were excluded from the sustainability assessment. Two facilities in DKK were found to be unsuitable as one facility was an outreach service, not in operation for all working days, and the other facility was attached to a hospital and performing outpatient services for hospital clients. One facility in WRH could not be visited, as the facility was closed for an extended period because of community protests. In BBR, two facilities were excluded, as the infrastructure of the facilities was not conducive to the implementation of the ICDM.

### Data collection and analysis

The principal investigator conducted an onsite inspection together with the operational managers, PHC clinic supervisors or local area managers, and the designated ICDM nurse at each facility to assess the implementation of the ICDM and identify factors affecting the sustainability. On completion of the *in situ* inspection, the operational manager and ICDM nurse were provided with an explanation and requested to complete the sustainability assessment tool. Data were collected between 12 August and 9 September of 2014.

## Sustainability model assessment tool

The NHS Institute for Innovation and Improvement SM self-assessment tool was utilised to assess the sustainability of the quality improvement process. The SM tool aims to enable teams to recognise and self-assess against key variables in their local context that determine whether a new practice is likely to be sustained and to prompt timely action to increase the likelihood of this being achieved.^17^ The SM details 10 key factors that increase the likelihood of sustainability and continuous improvement.^17^ The factors are grouped into three domains entitled ‘process’, ‘staff’ and ‘organisation’ (Table 2). The model was developed using information gathered from a review of management literature related to sustainability and research involving project leaders, directors, clinicians and global health care experts within a national improvement programme. Initially, over 100 factors were considered as being important ingredients for sustaining change. Through focus groups and other means, 250 NHS staff and health care experts were asked to rank these factors from 1 to 10, and from this, the final 10 factors were derived.^17^ The factors are grouped into three domains entitled ‘process’, ‘staff’ and ‘organisation’ (Table 2). For each of the 10 factors, respondents choose one of four statements they feel represent the ‘best fit’ with their current situation. The model developers used the data obtained from the key experts and conducted regression analyses to derive a weighted numerical score for each level of each factor, with the staff domain perceived as most important (52% of total weight), followed by ‘process’ (31%) and ‘organisation’ (17%).^17^ The component scores from each of these domains were added according to the master scoring guidelines of the SM model^18^ to obtain component and total scores. A score greater than 55 was indicative of sustainability of the improvement (ICDM) process. The one-way ANOVA was used to determine statistical significance (*p* < 0.05) between the three districts.

**TABLE 2:** Sustainability model criteria for sustainability.^20^

Description of variable	Maximum score
**Process**	**31.5**
Benefits beyond helping patients – Does the change reduce waste, duplication and added effort?	8.7
Credibility of evidence – Are the benefits to staff patients and organisation visible?	9.1
Adaptability of improved process – Does the change rely on an individual, group of people or finances to keep it going?	7
Effectiveness of system to monitor progress – Is special monitoring required?	6.7
**Staff**	**52.5**
Staff involvement and training to sustain the change – Play a part in implementation and design.	11.5
Staff behaviour to sustaining change – Staff inputs.	11
Senior leadership engagement – Are they involved and promote it?	15
Clinical leadership engagement – Are they involved and promote it?	15
**Organisation**	**16.9**
Fit with organisation strategic aims and culture – Is the change aligned to organisation strategic aims?	7.2
Infrastructure for sustainability – Staff facilities and equipment to sustain change.	9.7
**Maximum score**	**100.9**
**Minimum sustainability score**	**55**

*Source*: NHS Institute for Innovation & Sustainability

### Ethical consideration

The Biomedical Research and Ethics Committee (BREC) of the University of KwaZulu-Natal provided ethical approval for the study (BE: 423/13). Informed consent was obtained from operational managers and ICDM designated nurse prior to conducting the implementation and sustainability assessment.

## Results

### Total sustainability scores

Bushbuckridge had the highest mean sustainability score of 71.79 (95% CI: 63.70–79.89) followed by West Rand Health District (70.25 (95% CI: 63.96–76.53)) and Dr Kenneth Kaunda District (66.50 (95% CI: 55.17–77.83)) (Table 3). The mean sustainability scores across the three districts were not statistically significant (*p* > 0.05). Four facilities (11%) (one facility in BBR, one facility in WRH and two facilities in DKK) had an overall sustainability score of less than 55.

**TABLE 3:** Overall and component sustainability scores.

Variable	DKK	WRH	BBR
Benefits	8.7 (95% CI: 8.7–8.7)	8.39 (95% CI: 7.75–9.03)	8.20 (95% CI: 7.00–9.32)
Credibility	7.7 (95% CI: 6.8–9.0)	6.83 (95% CI: 5.07–8.59)	7.06 (95% CI: 7.06–9.29)
Effectiveness	4.66 (95% CI: 3.5–6.7)	5.65 (95% CI: 4.71–6.60)	6.28 (95% CI: 5.66–6.89)
Adaptability	3.03 (95% CI: 2.6–3.4)	3.14 (95% CI: 2.59–3.68)	3.21 (95% CI: 3.00–3.43)
Process	24.09 (95% CI: 21.9–27.4)	24.02 (95% CI: 20.90–27.13)	25.82 (95% CI: 23.21–28.43)
Staff involvement	9.03 (95% CI: 7.7–12.1)	9.07 (95% CI: 7.21–10.93)	10.37 (95% CI: 8.65–12.08)
Staff behaviours	4.46 (95% CI: 4.8–5.2)	5.1 (95% CI: 5.1–5.1)	4.46 (95% CI: 3.53–5.39)
Senior leadership	12.09 (95% CI: 7.5–16.9)	13.49 (95% CI: 11.37–15.62)	11.90 (95% CI: 8.86–14.94)
Clinical leadership	5.86 (95% CI: 3.4–7.6)	6.61 (95% CI: 6.42–6.80)	6.21 (95% CI: 5.31–7.10)
Staff	31.44 (95% CI: 23.8–41.4)	34.27 (95% CI: 30.76–37.77)	32.94 (95% CI: 27.03–38.84)
Organisational fit	5.28 (95% CI: 4.7–7.4)	5.15 (95% CI: 4.00–6.29)	6.48 (95% CI: 5.66–7.30)
Infrastructure	5.70 (95% CI: 3.4–8.4)	6.82 (95% CI: 4.52–9.11)	6.56 (95% CI: 4.51–8.61)
Organisation	10.98 (95% CI: 8.7–15.2)	11.96 (95% CI: 8.98–14.94)	13.04 (95% CI: 10.61–15.46)
**Total sustainability score**	**66.5 (95% CI: 55.52–78.85)**	**70.25 (95% CI: 63.96–76.53)**	**71.79 (95% CI: 63.70–79.89)**
**No. of facilities with scores below sustainability standard**			
Process	0	1	1
Staff	4	3	3
Organisation	2	2	3
Overall sustainability	2	1	1

BBR, Bushbuckridge; DKK, Dr Kenneth Kaunda District; WRH, West Rand Health District.

The process component of the SM tool obtained the highest proportionate sustainability score across all the three districts. Bushbuckridge had the highest mean process component score of 25.82 (95% CI: 23.81–28.43) followed by DKK with a mean score of 24.09 (95% CI: 20.88–27.29) and WRH with a mean of 24.02 (95% CI: 20.90–27.13). In DKK, all eight facilities achieved a score greater than the minimum score (17.33; 55%) for sustainability of the process component, whereas in BBR and WRH one facility each did not achieve the minimum score (17.33; 55%) for sustainability of the process component.

The mean organisational component score of 13.04 (95% CI: 10.61–15.46) was the highest in BBR, followed by 11.96 (95% CI: 8.98–14.94) in WRH and 10.98 (95% CI: 7.37–14.59) in DKK. Ten facilities (three in BBR, four in DKK and three in WRH) scored below the minimum score (9.30; 55%) for sustainability of the organisational component.

The staff component achieved the lowest proportionate sustainability score. WRH had the highest mean sustainability score for the staff component of 34.27 (95% CI: 30.76–37.77), followed by BBR with a mean score of 32.94 (95% CI: 27.03–38.84) and DKK with a mean score of 31.44 (95% CI: 22.13–40.75). Seven facilities (three in BBR, two in WRH and two in DKK) scored below the minimum score (28.88; 55%) for sustainability of the staff component.

## Subcomponent scores and performance

**FIGURE 2:**
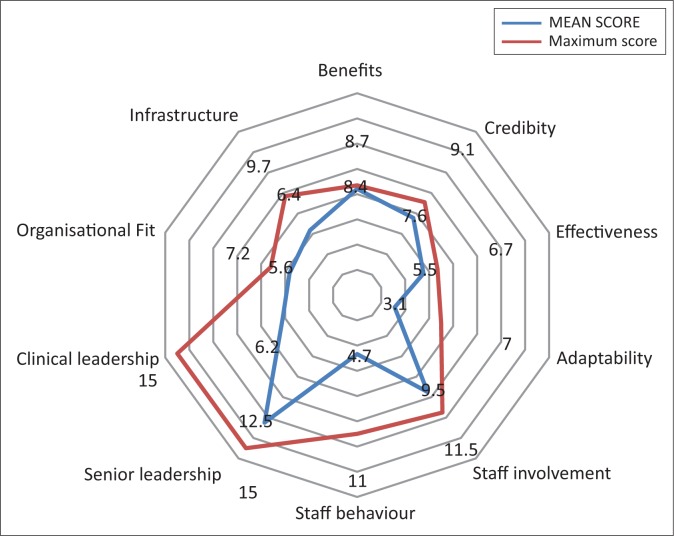
Sustainability mean component scores across the three districts.

The perceived benefit of the ICDM beyond helping the patient, credibility of evidence in favour of the ICDM, senior leadership involvement in the ICDM, staff involvement in design and implementation of the ICDM, the alignment of the ICDM with the organisational fit and the effectiveness of the ICDM were the strongest factors that promoted sustainability across all the three districts (Figure 2). The four main weaknesses of the ICDM implementation process that inhibit the sustainability across all the three districts include the less than optimal involvement of clinical leadership (doctors and senior professional nurses), staff negative behaviour towards the ICDM, adaptability or flexibility of the model to adapt to external factors and infrastructure limitations (Figure 2). Although infrastructure limitations emerged as an inhibiting factor, this was not prevalent across all the facilities in the districts.

## Discussion

There is a paucity of studies that have assessed the sustainability of complex health interventions in a developing country context. In the opinion of the authors, this study is the first to be conducted in South Africa assessing the implementation and sustainability of a complex health intervention implemented at a primary level. Furthermore, this study is the first to apply the NHS Institute for Innovation and Improvement SM tool to measure sustainability in a developing country context. The SM is a diagnostic tool that identifies strengths and weaknesses in the implementation plan and predicts the likelihood of sustainability of the quality improvement initiative.^19^ Although the tool was developed in the UK, inputs were obtained from global experts in its development. The simple nature of the tool with easy-to-understand components facilitated its application in the South African context.

Sustainability is a dynamic process and is achieved when health care professionals adapt the new working methods as routine methods.^20^ The literature indicates that partial sustainability was more common than continuation of the entire programme or intervention, even when full implementation was initially achieved.^13^ Therefore, it was important that this assessment was conducted to inform policy-makers on potential weaknesses in the implementation plan that could affect the rapid scale up and sustainability of the ICDM. Sustainability is affected by numerous intrinsic and extrinsic factors and cannot necessarily be dichotomised by a single value. However, for the purposes of the application of the SM tool, sustainability means the continuation or the integration of new practice within an organisation whereby it has become a routine part of care delivery and continues to deliver desired outcomes.

The implementation of the ICDM is a complex process involving interventions at multiple levels of the health system with a diverse range of stakeholders. The initial implementation was externally facilitated, and the ICDM activities for facility reorganisation were introduced in a phased approach using learning sessions and action periods of the breakthrough series^19^ to facilitate continuous quality improvement.

Although the decision to rapidly scale up the ICDM is commendable for getting research into policy and practice, it is important that after the initial impetus and enthusiasm, the implementation is sustained. The implementation of unsustainable programmes is expected to cause frustration and increase resistance to future improvement initiatives amongst already overburdened staff, and quality improvements in care delivery that are not sustained are a waste of resources.^15^

The facility reorganisation component of the ICDM and the clinical support provided to professional nurses in management of patients has supported the implementation and is predictive of sustainability as is evident from the sustainability scores in the process and organisation component of the SM model. A similar finding is reported from a study conducted across 22 Dutch disease management programmes that showed the quality of chronic care delivery (integration of care components, delivery system design, decision support and community linkages) during both the first and second year after programme implementation predicted the sustainability of these programmes.^15^ The demographic characteristics of participating professionals, such as age, gender and educational level, had no predictive effect on programme sustainability.^15^

Our study has shown four potential areas that may negatively affect the scale-up and sustainability of the ICDM staff behaviour towards change, clinical leadership, adaptability and infrastructure. These findings are consistent with those of an empirical literature review that indicated the stability of the workforce and attributes of the workforce, such as their skills and attitudes, are associated with the sustainability of a newly introduced programme.^13^ The support or participation of key stakeholders and funding were also regarded as important influences of sustainability.^13^

Although staff factors overall have scored above the minimum score for sustainability, the mean proportionate scores for staff factors were lower compared with process and organisation factors. Research has shown that many quality improvement programmes fail to become part of the habits and routines of professionals and that changing only the system of care delivery (integration of care components, delivery system design) will not change old working habits of professionals and may inhibit the sustainability of a quality improvement initiative.^15^

The low sustainability score for clinical leadership in our study can be attributed to the following factors: (1) Professional nurses who were previously employed on the vertical HIV programme were initially resistant to the improvement efforts in order to protect their autonomy and were negative about the impact that the ICDM would have on their workload^21^; (2) The implementation of the ICDM that focused on the professional nurse, with a peripheral role for the medical practitioner. The artificial boundaries created inadvertently by the nature of the implementation that the ICDM was a nurse-driven process further precipitated resistance especially of medical professionals working on the HIV programme in actively engaging with the ICDM implementation^22^; (3) The PHC supervisors or local area managers who were deemed to be the custodian of the implementation, mentoring and support faced numerous, complex, competing clinical and organisational demands^23^ that did not permit them to provide adequate leadership to the facilities.

The findings of our study in terms of adaptability indicate that although the process can be adopted and bring about organisational change, the optimal performance would be disrupted if the key individuals (operational managers and designated ICDM nurses) resigned or were redeployed. Findings from a study from the health care foundation show that team instability because of resignation and rotating staff between facilities because of staff shortages can result in stalled progress making it difficult to sustain collective knowledge and enthusiasm and negatively affecting the adaptability of the ICDM implementation.^23^

The fourth main challenge is that of infrastructure. Many of the facilities have outgrown their original design in terms of services and the population they serve. This is in part due to the original architecture as well as the expanding burden of disease. The infrastructure limitations have been highlighted because the process of care was not adequately mapped out in the facilities. However, infrastructure is an external limitation, and it is anticipated that with the successful implementation of the assisted self-support management component of the ICDM, the volume of patients attending the facilities will decrease.

### Study limitation

Although due diligence was exercised in maintaining the scientific integrity of the study, the study had numerous limitations.

Firstly, the study evaluated the implementation of the activities and sustainability from the health service perspective and did not investigate patient-related factors that may affect the continuous sustainability of the model. Furthermore, in assessing the sustainability only those professional nurses active in the implementation were surveyed and not other staff at the facility.

This study was concerned with the activities and did not objectively measure the outcomes of the implementation and their impact on the sustainability of the ICDM.

In addition, the data collection tool was self-administered and the potential of reporting bias cannot be excluded.

## Conclusion

The findings of this study indicate that the facility reorganisation and clinical support component contributed to a high sustainability score for the organisation and process component of the SM. The perceived poor adaptability, staff behaviour towards change, active participation of medical practitioners and infrastructure limitations have a negative impact on the sustainability of the ICDM implementation.

In rapidly scaling up the ICDM and to ensure sustainability, it is important that there is alignment between all programmes on the vision of integrated care. Change management, organisation support in terms of mentoring, supervision and addressing resource challenges together with the system of care changes have the ability to positively influence the behaviour of health professionals and thereby influence them to adopt the new method of practice as routine sustainability.^15^

A repeat assessment of the sustainability of the ICDM yearly should be conducted to enhance the value of the implementation.
